# Pharmacokinetic and Pharmacodynamic Properties of a Micro-Dose Nasal Spray Formulation of Desmopressin (AV002) in Healthy Water-Loaded Subjects

**DOI:** 10.1007/s11095-019-2628-1

**Published:** 2019-04-29

**Authors:** Karl-Erik Andersson, James Longstreth, Benjamin M. Brucker, Lysanne Campeau, Linda Cheng, Leo Francis, Seymour Fein

**Affiliations:** 1 Institute for Regenerative Medicine, Wake Forest University School of Medicine, Richard H. Dean Biomedical Building, 391 Technology Way, Winston-Salem, North Carolina USA; 2 Longstreth & Associates Inc, Mundelein, Illinois USA; 3 Department of Urology, Langone Health, New York University, New York, New York USA; 4 Division of Urology, Jewish General Hospital, McGill University, Montreal, Quebec, Canada; 5 Serenity Pharmaceuticals LLC, Milford, Pennsylvania USA; 6 Avadel Specialty Pharmaceuticals plc, Chesterfield, Missouri USA

**Keywords:** bioavailability, antidiuresis, urine osmolality, hyponatremia, safety

## Abstract

**Purpose:**

Antidiuretic therapy with desmopressin for nocturia has been hampered by formulations with high doses, low bioavailability and variable pharmacokinetics. AV002 (SER120), a novel, emulsified, microdose desmopressin nasal spray, with a permeation enhancer (cylcopentadecanolide), was developed to have pharmacokinetic characteristics suitable for nocturia treatment.

**Methods:**

Twelve healthy subjects participated in an open-label, dose-escalating study. Water-loaded subjects were sequentially dosed every 48 h with AV002 0.5, 1.0, 2.0 μg and 0.12 μg desmopressin subcutaneous (SC) bolus injection.

**Results:**

AV002 intranasal administration produced a time-to-maximum concentration (T_max_) between 15 and 30 min and a maximum concentration (C_max_) <10 pg/mL. C_max_ and area under the curve showed dose proportionality. Coefficient of variation for AV002 was similar to that observed for the SC dose. Bioavailability of AV002 was approximately 8% compared to SC injection. AV002 demonstrated pharmacodynamic effects within 20 min of dosing and showed increasing magnitude and duration with escalating doses. AV002 2.0 μg had maximum median urine osmolality of 629 mOsm/kg and median urine output ≤2 mL/min for 5–6 h.

**Conclusions:**

AV002 demonstrated rapid absorption, high bioavailability, limited duration of action, and low coefficient of variation, suggesting it may be a suitable formulation for nocturia treatment.

**Trial registration** not required (single-center, phase 1).

## Introduction

The vasopressin analogue, desmopressin, is a synthetic nonapeptide originally introduced for treatment of central diabetes insipidus (1–3) and later in patients with primary nocturnal enuresis (4,5) and nocturia caused by nocturnal polyuria (6–8). Similar to natural antidiuretic hormone (arginine vasopressin), the antidiuretic activity of desmopressin is mediated by binding to the vasopressin receptor 2 on principal cells of renal collecting tubules, thereby stimulating a cascade of biochemical events that increases urine concentration and decreases urine production (9). These changes are mediated by passive reabsorption of solute-free water down the osmotic gradient created by the hypertonicity in the renal medulla (9).

Therapeutic use of desmopressin for antidiuretic purposes includes both intranasal and oral formulations. The most important clinical issue of current marketed desmopressin products is the potential to develop hyponatremia, which is dose-dependent and can occur in adults of all ages but is far more prevalent in patients age 50 and older (10). A low-dose desmopressin formulation, with high bioavailability and low coefficient of variation that can achieve consistent peak blood concentrations and a predictable and limited duration of effect, is important for the safe and effective treatment of nocturia. A pharmacokinetic profile limiting the duration of the antidiuretic effect to approximately 4 to 6 h during sleep is desirable.

The pharmacokinetics of desmopressin have been characterized by several investigators. Although some studies have been performed in normally hydrated subjects (11,12), most studies investigating the antidiuretic activity of desmopressin in healthy human subjects have been performed in orally water-loaded subjects (13–17) with high doses of the drug. But, pharmacodynamic and pharmacokinetic information on very low-doses of intranasal desmopressin preparation is currently lacking. Given that very low doses of intranasal desmopressin as an antidiuretic treatment for patients with nocturia may mitigate the risk of hyponatremia, the pharmacodynamic and pharmacokinetic profiles of this formulation are clinically relevant. The main objective of this phase I study was to investigate the antidiuretic activity of different dosages of a new, emulsified micro-dose desmopressin formulation (AV002) and secondarily, its pharmacokinetic profile when administered intranasally to water-loaded healthy subjects, and to compare the AV002 effects to a bolus subcutaneous (SC) injection of desmopressin.

## Materials and Methods

This was an open-label, escalating dose titration study. The study protocol and any amendments were reviewed by PRACS Institutional Review Board/Ethics Committee and the study was conducted in accordance with the ethical principles in the Declaration of Helsinki, and in compliance with the approved protocol, good clinical practice and applicable regulatory requirements, including informed consent. Trial registration was not required (single center, phase I study).

### Subjects

Six male and 6 female non-smoking healthy subjects, 18 to 40 years of age and with a body mass index (BMI) between 20 and 32 kg/m^2^ for males and between 20 and 28 kg/m^2^ for females, were recruited. Screening evaluations, performed within 14 days of the first treatment, included medical history, physical examination, nasopharyngeal examination, electrocardiogram (ECG), complete blood count with differential, clinical chemistries including serum osmolality, urinalysis including urine osmolality, urine drug screen, serology for HIV, hepatitis B surface antigen and hepatitis C antibody and for female subjects, serum pregnancy test.

On Day −1, 14 eligible subjects (12 enrolled and 2 backup subjects) were admitted to the study center and given an abbreviated physical exam including nasopharyngeal examination, vital signs, a test for alcohol, serum sodium and osmolality and urine osmolality assessments. Female subjects were given a repeat serum pregnancy test.

### Hydration

Subjects were water-loaded prior to dosing with AV002. The water-loading methodology required subjects to drink 1 to 2 l of water between 7 PM and 9 PM on Day −1 to prepare for the water-loading process the following morning. Thereafter, they were able to drink ad libitum until the start of the water-loading process the next morning. Subjects fasted from food starting at 8 PM the night before dosing until breakfast on the day of dosing. To achieve the water-loaded state and inhibit the secretion of endogenous vasopressin so that the antidiuretic effect observed would be entirely attributable to administered desmopressin, subjects were required to drink a volume of water corresponding to 1.5% to 3% of their body weight. The initial water load of 1.5% was ingested over approximately 30 min. If this did not achieve a minimum urine output of 10 mL/min, subjects ingested another 1.5% of body weight in water during the second hour to achieve water-loaded state (exceeded 10 mL/min in 2 consecutive urine output measurements) (16,18–22). Subjects were asked to void every 20 min and urine output was replaced with an equal volume of ingested water to maintain a constant water-loaded state throughout the observation period (8 h) for each dose.

### Dosing

Each subject was dosed 4 times over 1 week with dosing occurring every other day. Doses of AV002 were administered at 0.5, 1.0, and 2.0 μg intranasally. Desmopressin acetate for SC injection was administered in the left or right upper arm at a dose of 0.12 μg, which was selected from pre-clinical bioavailability study results.

On Days 1, 3 and 5, subjects were dosed intranasally with escalating doses of AV002 nasal spray. On Day 7, 6 subjects were given a single SC bolus injection of desmopressin. Pharmacodynamic and pharmacokinetic metrics were evaluated at each dose level.

### Desmopressin Formulations

AV002 was supplied by the sponsor in 10 mL amber glass bottles with each bottle containing 5 mL of AV002 nasal spray emulsion at a concentration of 5 μg/mL of desmopressin. Each milliliter of the formulation also contained the following excipients: cyclopentadecanolide (CPD, a permeation enhancer), cottonseed oil, sorbital monolaurate, polysorbate 20, anhydrous citric acid, sodium citrate dihydrate and purified water, USP for injection. The particle size distribution of the AV002 nasal spray has average diameters of 0.8 μm. 1.3 μm and 2.3 μm for 10%, 50% and 90% of the particles respectively in this formulation.

Desmopressin acetate for injection (4 μg/mL in 10 mL vial) was obtained as a commercial product and a 1:5 dilution was prepared with 0.9% Sodium Chloride USP for Injection as a diluent to reach a final concentration of 0.8 μg/mL. This diluted desmopressin acetate solution was injected subcutaneously at a dose of 0.12 μg in a volume of 150 μL. The preparation of the diluted desmopressin solution was done within 1 h of dosing.

### Measurements

Urine output and urine osmolality were assessed every 20 min up to 8 h post-dosing and hourly thereafter until the subject’s urine output returned to baseline (exceeded 10 mL/min on 3 consecutive 20-min measurements), or exceeded 10 mL/min in an hourly collection, or the urine osmolality was <200 mOsm/kg on one occasion. Urine specific gravity was measured at −20 min, 0 h and at hourly intervals up to 8 h post dose. Serum sodium and osmolality was measured prior to dosing (0) and at 2, 4, 6 and 8 h post dose. Blood pressure and pulse rate were recorded at the start of the water-loading process and at 30-min intervals until the first scheduled time point after the end of the water-loading process to determine if there was a fall in arterial blood pressure indicating endogenous vasopressin release. Blood sampling for pharmacokinetic determinations was performed pre-dose (0), and at 5, 10, 15, 30, 45, 60, 90, 120, 180, 240, and 360 min post dose.

Three meals were served each day with calorie and sodium content approximately equal in all meals. Breakfast was served prior to water-loading on Days 1, 3, 5, and 7. On Day 2, subjects remained in the clinic and had vital signs measured. They were encouraged to drink 1 to 2 l of water between 7 PM and 9 PM. Thereafter, they were to drink fluid ad libitum until the start of the water-loading process on Day 3 and were required to remain fasted from food from 8 PM until breakfast the next morning. Day 3 and 4 procedures followed those of Days 1 and 2, respectively, except 2 nasal sprays were administered for a nominal dose of 1.0 μg of desmopressin on Day 3. Similarly, Days 5 and 6 procedures were the same as Days 1 and 2 except the dose of test drug was 4 nasal sprays, equivalent to a nominal dose 2.0 μg of desmopressin. On Day 7, 3 male and 3 female subjects were randomized to receive 1 single SC bolus injection of desmopressin solution (150 μL of 0.8 μg/mL solution equivalent to a dose of 0.12 μg of desmopressin). All other procedures were identical to Day 1. On Day 8, the subjects underwent an exit physical examination with vital signs and clinical laboratories (hematology, chemistries, urinalysis).

### Desmopressin Assay

Plasma samples were analyzed using a validated radioimmunoassay assay (Celerion Switzerland AG) for desmopressin with a lower limit of quantification (LLOQ) of 2.5 pg/mL and upper limit of quantitation of 160 pg/mL. A 1.25 pg/mL calibration standard was included in the assay runs and concentrations between 1.25 and 2.5 pg/mL were reported out even though they were below the nominal LLOQ. Mean precision values for the low, medium and high-quality control samples included in the accepted assay runs were 6.7% to 11.9%, and mean accuracy values ranged from −9.0% to +0.1%.

### Statistics

The statistical analysis was descriptive in nature and presented as means, medians, and ranges for continuous variables and as numbers and percentages for categorical variables. All individual subject data were listed as measured. All significance tests were 2-sided, and all confidence intervals were presented with 95% degree of confidence.

Primary pharmacodynamic endpoints were urine osmolality and urine output at specified time points prior to and after dosing with escalating doses of nasal spray and with SC bolus injections of desmopressin.

Pharmacokinetic variables were generally analyzed using model-independent methods. A single compartment model with first order absorption was used to qualitatively assess the impact of probable pharmacokinetic behavior of desmopressin when concentrations declined below the assay lower limit of quantification.

Pharmacokinetic calculations were performed using built in functions in the Excel 2003 component of Microsoft Office 2003 (Microsoft, Redmond, WA). The correctness of the calculation techniques was confirmed by comparison to output from WinNonlin 4.0.1 (Pharsight Corporation, Sunnyvale, CA). Summary statistics, graphs and figures were generated using built in functions and features of Excel 2003. Standard deviation (SD) and standard error of the mean (SEM) were displayed to the same number of decimal points as the mean value. Coefficients of variation were displayed as percentages to one decimal place.

## Results

### Demographics

The subjects enrolled in the study had a mean age of 22.3 years and 91.7% were Caucasian. The mean BMI was 24.5 kg/m^2^ and ranged between 21 and 30 kg/m^2^. There were no clinically meaningful findings at screening in vital signs, physical examination, clinical laboratory values, ECGs, or concomitant medications in any of the subjects enrolled in the study.

### Pharmacokinetics

Pharmacokinetic samples were collected at pre-dose and 0.083, 0.167, 0.25, 0.50, 0.75, 1.05, 1.50, 2.05, 3.05, 4.05, and 6.05 h post dose each dosing day. The samples collected from Day 1 after AV002 nasal spray administration of 0.5 μg doses were not assayed because results at the higher doses suggested that blood concentrations at the 0.5 μg dose would be almost entirely below the LLOQ of the assay. The plasma pharmacokinetics of desmopressin following AV002 intranasal administration at doses of 1.0 and 2.0 μg, and SC injection of 0.12 μg of desmopressin, were evaluated (data for 2 μg are shown in Table I). Systemic absorption of AV002 was rapid and peak concentrations were reached in 20 to 65 min. Maximum concentration (C_max_) and area under the curve (AUC) increased with increasing doses of AV002 nasal spray in a dose-proportional manner. The mean C_max_ for the 2.0 μg dose was 6.24 (± SD 2.25) pg/mL, ranging from 1.59 to 10.1 pg/mL, with a coefficient of variation of 36.0%, comparable to SC injection (Table I). The mean C_max_ for the 1.0 μg dose was 2.79 (±1.44) pg/mL, ranging from 1.49 to 4.89 pg/mL with a coefficient of variation (CV) of 51.6%. The mean AUC for AV002 1.0 μg nasal spray dose was 6.49 (±3.59) pg•h/mL, 11.5 (±7.9) pg•h/mL for AV002 2.0 μg nasal spray dose; and 10.2 (±4.9) pg•h/mL for the 0.12 μg desmopressin SC injection.

**Table I:** AV002 Intranasal Spray and Desmopressin Subcutaneous Injection Pharmacokinetic Metrics

PK Parameter	AV002 2.0 μg Intranasal Mean ± SD (CV%)	Desmopressin 0.12 μg Subcutaneous Mean ± SD (CV%)
C_max_ (pg/mL)	*N* = 12	*N* = 6
	6.24 ± 2.25	2.77 ± 0.98
	(36.0)	(35.4)
T_max_ (h)	*N* = 12	*N* = 6
	0.354 ± 0.188	0.883 ± 0.349
	(53.2)	(39.5)
AUC_∞_ (pg.h/mL)	*N* = 8	*N* = 3
	11.5 ± 7.9	10.2 ± 4.9
	(68.6)	(47.5)
T_1/2_ (h)	*N* = 8	*N* = 3
	1.33 ± 0.56	2.09 ± 0.32
	(42.3)	(15.4)

*AUC* area under the curve, *C*_*max*_ maximum concentration, *CV* coefficient of variation, *PK* pharmacokinetic, *SD* standard deviation, *T*_*1/2*_ half-life, *T*_*max*_ time to maximum concentration

The mean half-life (T_½_) was 1.13 h and 1.33 h for AV002 1.0 and 2.0 μg nasal spray doses, respectively, and was 2.09 h for the 0.12 ng SC desmopressin dose. The systemic bioavailability of the 1.0 μg nasal spray dose could not be estimated but the 2.0 μg dose was 7.4 ± 2.3% that of the SC injection, which was used as a reference.

The apparent clearance of the AV002 1.0 μg and 2.0 μg intranasal doses (184 ± 83 L/h and 232 ± 118 L/h, respectively) was higher than that of the SC desmopressin dose (13.6 ± 6.0 L/h), and showed no indications of a depot or sustained-release effect. The 2.0 μg intranasal dose had a mean time to maximum concentration (T_max_) of 21 min and the mean T_max_ for SC bolus injections was 53 min (Table I).

### Pharmacodynamics

**Fig. 1:**
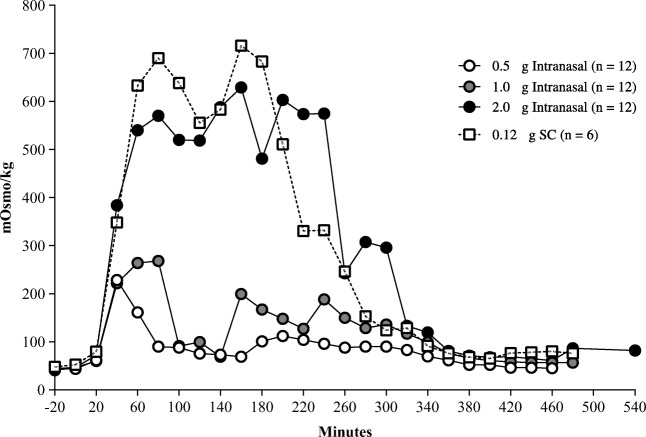
Median urine osmolarity over time in healthy volunteers who received intranasal AV002 or subcutaneous desmopressin.

Pharmacodynamics for AV002 intranasal doses and desmopressin injection were analyzed in all 12 enrolled subjects who received the preparation. Figure 1 shows the dose-response relation in terms of the magnitude of increase in urine concentration from baseline for all subjects. There was a rapid onset of increase in urine osmolality at all dose levels with onset occurring within 20 min after dosing. The maximum median urine concentration (mOsm/kg) at each of the 3 doses of AV002 nasal spray (0.5 μg, 1.0 μg and 2.0 μg) was dose-dependent, occurred between 40 and 160 min after dosing and ranged from 229 to 629 mOsm/kg. For the SC desmopressin dose, a maximum value of 716 mOsm/kg was recorded at 160 min post dosing.

**Fig. 2:**
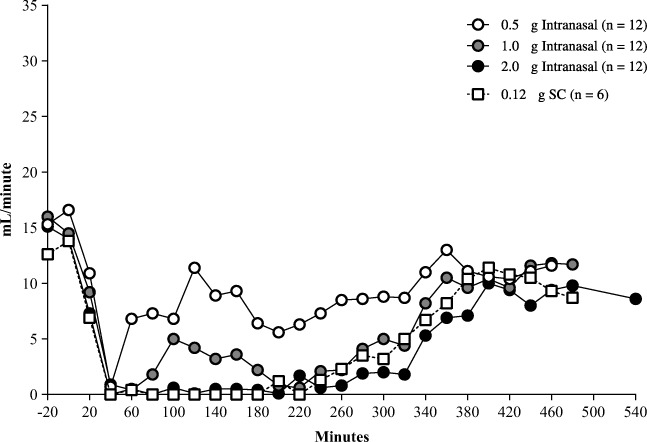
Median urine output over time in healthy volunteers who received intranasal AV002 or subcutaneous desmopressin.

The antidiuretic effect of AV002 nasal spray preparation resulted in increases in urine osmolality and decreases in urine output. The decrease in urine output had a rapid onset occurring within 20 min after dosing. The median magnitude and duration of the decrease was approximately dose proportional across the 3 nasal spray doses studied. The 0.12 μg desmopressin SC dose produced effects on urine output similar to the 2.0 μg AV002 nasal spray dose in terms of magnitude and duration of action. The 1.0 μg dose of AV002 nasal spray maintained decreased urine output even in the water-loaded state at approximately 4 mL/min or less for 4 to 5 h; the 2.0 μg dose and the 0.12 μg SC injection maintained decreased urine output at approximately 2 mL/min or less in the water-loaded state for 5 to 6 h and 4 to 5 h, respectively. Urine output was inversely related to mean urine osmolality. The urine output profile over time with AV002 2.0 μg nasal spray dose was similar to the profile for 0.12 μg SC desmopressin (Fig. 2).

### Safety

All 12 subjects received a single dose of treatment on Study Days 1 (0.5 μg AV002), 3 (1.0 μg AV002), 5 (2.0 μg AV002) and 7 (0.12 μg SC desmopressin injection). The most common adverse events (AEs) were headache in 3 subjects, nasal discomfort in 3 subjects and pain in an extremity in 3 subjects. All AEs were transient and self-limited and did not require treatment or other medical intervention. No severe or serious AEs occurred. No subject discontinued the study because of an AE.

There was no apparent relationship between dose level and AEs, and there were no clinically meaningful changes in clinical laboratory results including hematology, chemistries and urinalysis.

Following the decreases in serum sodium secondary to water-loading at baseline, post treatment serum sodium for all administered doses remained stable while maintaining water-loading in a balanced fluid intake and output fashion. There were no clinically meaningful changes or abnormalities from baseline for vital signs, physical examination or 12-lead ECG findings.

In most subjects, serum sodium stabilized from the pre-dose baseline value after test drug was administered, and in some cases modestly increased over time as was expected with the balanced input and output methodology of the study following the water-loading procedure (Table II).

**Table II:** Mean Serum Sodium at Pre-dose and During Treatment, and Change from Pre-dose in Water-Loaded Subjects by Treatment Group

	Mean Serum Sodium (mmol/L)				Change from Pre-Dose (mmol/L)			
	AV002 Nasal Spray			SC	AV002 Nasal Spray			SC
	0.5 μg (*N* = 12)	1.0 μg (*N* = 12)	2.0 μg (*N* = 12)	0.12 μg (*N* = 6)	0.5 μg (*N* = 12)	1.0 μg (*N* = 12)	2.0 μg (*N* = 12)	0.12 μg (*N* = 6)
Pre-dose	136.7	133.7	132.0	134.3	−	−	−	−
2 Hr. Post Dose	134.2	130.2	131.4	130.7	−2.5	−3.5	−0.6	−3.6
4 Hr. Post Dose	135.6	131.6	132.0	130.5	−1.1	−2.1	0	−3.8
6 Hr. Post Dose	137.5	134.5	132.0	132.3	0.8	0.8	0.8	−2.0
8 Hr. Post Dose	138.6	135.8	134.7	134.2	1.9	2.1	2.7	−0.1

*Hr* hour, *SC* subcutaneous desmopressin injection

## Discussion

The results of the study revealed that AV002, a new formulation of micro-dose desmopressin nasal spray, produced rapid and substantial antidiuretic effects in water-loaded subjects with single doses of 0.5 μg, 1.0 μg and 2.0 μg. In this investigation, the main objective was to explore the pharmacodynamic effects of AV002 in terms of urine osmolality and urine output. The magnitude and duration of the antidiuretic effect increased with increasing dose. The doses of AV002 tested produced durations of antidiuresis in the 4 to 6 h range, as intended. AV002 had a rapid T_max_ of approximately 20 min, which was faster than that of the SC injection. C_max_ and AUC_∞_ showed dose proportionality and the estimated relative bioavailability was more than double that of the current high-dose desmopressin aqueous nasal sprays (~3.5% according to prescribing instructions) (23,24). Kohler *et al.* using high doses of desmopressin in healthy volunteers showed that the relative bioavailability of intranasal administration of aqueous nasal was approximately 2% of SC injection (25), The low coefficient of variation, comparable to that observed for the SC dose, suggests that AV002 will have a consistent pharmacokinetic profile from dose to dose and patient to patient, resulting in a predictable peak plasma concentration and duration of action. AV002 was safe and well tolerated at all doses tested. No drug-related hyponatremia was observed. The majority of AEs were mild; laboratory and physical findings were within normal limits or not clinically meaningful. Thus, AV002 nasal spray is expected to exert the required antidiuretic effect for the desired, limited duration and to be a safe and effective therapy for adult nocturia.

Studies in healthy subjects and in patients with cranial diabetes insipidus (26–28) have shown that dose and route of administration seem to affect the pharmacokinetics of desmopressin. In the present study, the 1.0 μg intranasal and 2.0 μg intranasal doses of AV002, and the 0.12 μg SC desmopressin dose, reached low pg/mL concentrations in plasma. Although many samples had desmopressin concentrations below the limits of detection, the antidiuretic pharmacodynamic effect of desmopressin was still measurable at plasma concentrations in the sub-picogram per mL range.

AV002 provided consistent delivery of drug to the intravascular compartment with a bioavailability relative to SC injection of 7% to 8% and a coefficient of variation comparable to that of SC for C_max_, T_max_ and AUC_∞_. This intranasal bioavailability value is substantially higher than the 3.4% observed by Fjellestad-Paulsen *et al.* (10) after intranasal administration of 20 μg of desmopressin in an aqueous formulation to healthy volunteers. The calculated bioavailability of AV002 in this study may be an underestimate because of the very low plasma concentrations achieved and the limitations of the assay in terms of the LLOQ. The rapid uptake and systemic availability of AV002 is likely aided by the emulsified formulation and the presence of the novel permeation enhancer CPD.

The elimination half-life in the present study (1.5 h) appeared to be similar to or even shorter than that reported in other studies of water-loaded subjects (14–16). It should be noted that the desmopressin doses given in previous studies were high compared with those of the present investigation, so generally stayed above the assay LLOQ for longer.

The short T_max_ of AV002 is indicative of the rapidity of absorption of drug through the nasal mucosa. Combination of the rapidity of absorption plus a short systemic half-life leads to a “rapid-on/rapid-off” antidiuretic pharmacodynamic profile. It is possible that the magnitude and duration of the antidiuretic effect of each of the micro-doses of desmopressin of AV002 administered in this study may have been underestimated because of the subjects’ water-loaded state. Intake of fluid increases excretion of water by the kidney. Inhibition of vasopressin secretion, which decreases the activity of renal sympathetic nerves or hormonal release, is a possible effector mechanism in this response (29). The suppression of vasopressin secretion is mediated not only by osmo- and baroreceptors, but also, stimulation of oropharyngeal receptors is probably involved (30). In the present study the median maximum urine osmolality after intranasal AV002 ranged from 229 to 629 mOsm/kg; after a 0.12 μg SC injection it reached 716 mOsm/kg. This is similar to the results presented by other investigators (16), the urine osmolality in euhydrated subjects reached up to 935 mOsm/ kg (11). Thus, it seems that the volunteers’ ability to concentrate urine in the present study was little affected by the water load. The changes observed in serum sodium, although clinically insignificant, were largely the result of the water-loading process and are unlikely to be relevant in euhydrated states.

## Conclusion

The present emulsified micro-dose desmopressin nasal spray formulation (AV002) with a permeation enhancer has pharmacokinetic and pharmacodynamic properties likely well suited for the treatment of patients with nocturia.

## Data Availability

The datasets generated during and/or analyzed during the current study are not publicly available but are available from the corresponding author or from Avadel Repository/Datahouse on reasonable request.

## Abbreviations

CPD: Cyclopentadecanolide
LLOQ: Lower limit of quantification
